# Nucleolar stress: Friend or foe in cardiac function?

**DOI:** 10.3389/fcvm.2022.1045455

**Published:** 2022-10-31

**Authors:** Daliang Yan, Lu Hua

**Affiliations:** ^1^Department of Cardiovascular Surgery, Taizhou People’s Hospital, Taizhou, China; ^2^Department of Oncology, Taizhou People’s Hospital, Taizhou, China

**Keywords:** nucleolus, nucleolar stress, cardiac disease, nucleostemin, nucleophosmin, nucleolin, senescence

## Abstract

Studies in the past decades have uncovered an emerging role of the nucleolus in stress response and human disease progression. The disruption of ribosome biogenesis in the nucleolus causes aberrant nucleolar architecture and function, termed nucleolar stress, to initiate stress-responsive pathways via nucleolar release sequestration of various proteins. While data obtained from both clinical and basic investigations have faithfully demonstrated an involvement of nucleolar stress in the pathogenesis of cardiomyopathy, much remains unclear regarding its precise role in the progression of cardiac diseases. On the one hand, the initiation of nucleolar stress following acute myocardial damage leads to the upregulation of various cardioprotective nucleolar proteins, including nucleostemin (NS), nucleophosmin (NPM) and nucleolin (NCL). As a result, nucleolar stress plays an important role in facilitating the survival and repair of cardiomyocytes. On the other hand, abnormalities in nucleolar architecture and function are correlated with the deterioration of cardiac diseases. Notably, the cardiomyocytes of advanced ischemic and dilated cardiomyopathy display impaired silver-stained nucleolar organiser regions (AgNORs) and enlarged nucleoli, resembling the characteristics of tissue aging. Collectively, nucleolar abnormalities are critically involved in the development of cardiac diseases.

## Introduction

Ribosome biogenesis is a highly conserved biological process essential to all living organisms (1). In eukaryotic cells, the nucleolus is a prominent sub-nuclear compartment central to ribosome biogenesis (2). The nucleolus is organized around ribosomal DNA (rDNA) distributed within acrocentric chromosomes. rDNA is transcribed into 47S pre-ribosomal RNA (pre-rRNA) by RNA polymerase I (Pol I) transcriptional machinery. Then, 47S rRNA is cleaved and processed into mature ribosomal RNAs, including 28 S, 18 S and 5.8 S rRNAs, *via* the assistance of heterogeneous nuclear ribonucleoproteins (hnRNPs) (3). Finally, the rRNAs were assembled, together with ribosomal proteins and 5S rRNA, into 40S and 60S ribosomal subunits, followed by the export of these subunits into cytoplasm. rRNA transcription is fundamental to nucleolar dynamics and integrity. Mammalian oocytes and zygotes contain morphologically distinct and transcriptionally inactive nucleoli termed nucleolus precursor bodies (NPBs), which consist of ribosomal proteins, fibrillarin and hnRNPs (4). rRNA transcription serves as a seeding mechanism to initiate nucleolar assembly (5, 6). Thus, the dynamics of nucleolar architecture is tightly controlled by the transcription of ribosomal RNA (rRNA) and the demand of ribosome synthesis.

Accumulating data have suggested a critical involvement of ribosome biogenesis and nucleolar dynamics in stress responses and human diseases. In this regard, nucleolar stress, a term indicating the disruption of ribosome biogenesis and nucleolar integrity, has been increasingly recognized as an important modulator of stress responses. Nucleolar stress is initiated under various stress conditions, including genotoxic stress, hypoxia, nutrient deprivation and thermal stress, and plays a critical role in stress-induced signaling transductions. Notably, data obtained from both clinical and basic researches have pointed to a pivotal role of nucleolar malfunction in the development of cardiac diseases. Of importance, nucleolar stress may elicit both beneficial and adverse effects during cardiac injury. In this review, we summarize current findings linking nucleolar function to cardiovascular diseases, and discuss both the protective and detrimental effects of the nucleolus and nucleolar stress.

## The non-canonical function of the nucleolus

While the canonical role of the nucleolus as a ribosome factory has been well-characterized, studies in recent years have suggested that the nucleolus possesses various non-ribosomal functions. The nucleolus is broadly implicated in the regulation of transfer RNA (tRNA) expression and processing, centromere assembly, nuclear architecture, X-chromosome inactivation and DNA damage responses (7–10). These studies indicated that, besides a central role in ribosome biogenesis, the nucleolus is critically involved in the regulation of signaling transduction and cellular homeostasis. Many nucleolar proteins and non-coding RNAs have been reportedly involved in various cellular responses and metabolic control. These studies highlight the importance of the non-canonical function of the nucleolus in the development of various human diseases, including cardiac damage and failure.

Multiple proteomic studies revealed that the nucleolus contains thousands of proteins, of which only a small proportion have been associated with ribosome biogenesis (11). The dynamics of nucleolar proteome and morphology are closely associated with cellular homeostasis and proliferative status (12). During cell cycle progression, the nucleolus is disassembled at the end of prophase as a result of nucleolar protein phosphorylation by Cdk1-cyclin B complex (13). The reassembly of the nucleolus during telophase is initiated by rRNA transcription and subsequent recruitment of various nucleolar proteins. Recent investigations revealed that the nucleolus is assembled through liquid–liquid phase separation (LLPS), displaying dynamic exchange with the surrounding nucleoplasm (14, 15). Nucleolar proteins may constitutively shuttle within and outside the nucleolus, and the dynamics of nucleolar proteins are regulated by metabolic and genotoxic stresses (16–18). Moreover, nucleolar accumulation of various proteins critical for intracellular signaling transduction and cell physiology has been increasingly characterized in the past decades (19). Mechanistic investigations revealed that proteins critical to signaling transduction may be sequestered in the nucleolus, especially under stress conditions. For instance, hypoxia and acidosis may trigger nucleolar sequestration of E3 ubiquitin ligase VHL, which is key to HIF-1α stabilization and the transcription of hypoxia-responsive genes (20, 21). Some recent studies uncovered that the nucleolus plays a central role in nuclear protein quality control in response to heat shock (22, 23). It was revealed that nuclear proteins, such as the Polycomb group protein (PcG) complex, may accumulate in the nucleolus under heat shock, and undergo recovery and refolding via an Hsp70-dependent mechanism after exiting from heat shock. Furthermore, mounting investigations suggested that the nucleolus serves as a compartment of protein ubiquitination and degradation. Nucleolar translocation of various nuclear proteins, including histones, cell cycle regulators and transcriptional factors, leads to their ubiquitination and proteasomal degradation (24–27). These findings highlight the importance of non-ribosomal functions of nucleolar proteins in cellular homeostasis.

In addition, small nucleolar RNAs (snoRNAs) have also been reportedly involved in various biological processes. snoRNAs are 60–300 nucleotide non-coding RNAs primarily localized in the nucleolus (28). snoRNAs play indispensable roles in rRNA chemical modifications, but also exert various non-ribosomal functions. Carlos Michel et al. identified three snoRNAs in ribosomal protein L13a (rpL13a) locus, including U32a, U33 and U35a, play critical roles in transmitting metabolic stress pathways (29). Further studies revealed that these snoRNAs modulate mitochondrial metabolism and the production of reactive oxygen species (ROS), leading to alterations in pancreatic insulin secretion and systemic glucose metabolism (30). Nuclear snoRNA may function as a component of spliceosome to regulate pre-mRNA splicing (31, 32). snoRNAs may also be released from the nucleolus into the cytoplasm under metabolic stress and doxorubicin exposure (33, 34). In the cytoplasm, snoRNAs may form mRNA-snoRNA to regulate mRNA translation and decay (35, 36). snoRNAs may also directly bind to various proteins to modulate the activation of signaling pathways (37, 38). Notably, altered expression of snoRNAs has also been observed in cardiomyopathic conditions, implicating an involvement of snoRNAs in cardiac disease progression (39–41).

## The nucleolus serves as stress sensor

Ribosome biogenesis is an energy-intensive process, and is thus subjected to delicate regulation by various upstream signals (42). Coordinate regulation of rRNA transcription is achieved mainly through modulating the activity of key components of Pol I transcriptional machinery by various upstream regulators (43). Pol I transcriptional machinery is a multi-subunit protein complex, composed of Pol I and various essential transcriptional co-factors, including SL1, TIF-IA and UBF (44). The expression and posttranscriptional modification of these proteins are tightly regulated by signaling pathways involved in cell proliferation, mitogenic growth, nutrient and energy sensing, as well as stress responses. For instance, TIF-IA is phosphorylated on Ser635 residue by AMPK following energy deprivation, and on Thr 200 residue by JNK2 following oxidative stress (45, 46). Both modifications cause the dissociation between SL1 and TIF-IA, consequently leading to the disruption of rRNA transcription. On the other hand, phosphorylation of TIF-IA by growth-related kinases, including EKR, mTOR and CK2 enhances TIF-IA activity and rRNA synthesis (47–49). Likewise, the activity of SL1, UBF and Pol I are also tightly regulated by posttranscriptional modifications, particularly phosphorylation, in response to upstream signals (43). In addition to the regulation of Pol I transcriptional machinery, epigenetic regulation of rDNA accessibility also plays a pivotal role in nucleolar dynamics (50). For instance, owing to open chromatin conformation and high rDNA accessibility, embryonic stem cells (ESCs) typically contain one large nucleolus. The exit from pluripotency in ESCs triggers epigenetic silencing of rDNA, leading to the formation of heterochromatic shell that compartments the large nucleolus into smaller nucleoli (51, 52). Notably, data obtained from multiple organisms revealed that epigenetic silencing of rDNA is tightly correlated with biological aging (53, 54). Thus, rRNA transcription and nucleolar morphology are closely associated with cellular physiological status.

Mounting studies have revealed that nucleolar integrity serves as a stress sensor and signaling hub following stress conditions (55). Nucleolar stress caused by aberrant rRNA transcription disrupts nucleolar integrity, leading to the aberrant release of nucleolar proteins, such as ribosomal proteins, NPM and NS, into the nucleoplasm, where they bind to various signaling proteins and alter their stability and activity. A most prominent stress pathway downstream of nucleolar stress is the p53 pathway. A pioneer work conducted by Carlos Rubbi and Jo Milner revealed that nucleolar disruption plays a causal role in p53 activation under DNA damage and other stress conditions, and that the disruption of nucleolar integrity is sufficient to activate p53 in the absence of stress stimuli (56). Later investigations uncovered that multiple nucleolar proteins, including NPM, p14*^Arf^*, ribosomal proteins RPL11 and RPL5, are key to p53 stabilization following nucleolar disruption (57). For instance, the blockade of rRNA transcription causes nucleoplasmic accumulation of 5S ribonucleoprotein particle (5S RNP), which comprises the 5S rRNA, ribosomal proteins RPL11 and RPL5 (58). This complex binds to and inactivates mouse double minute 2 homolog (MDM2), an E3 ligase responsible for p53 ubiquitination and degradation under quiescent conditions, leading to p53 accumulation and activation (59). Nucleolar stress-p53 pathway has been associated with a variety of cellular events, including senescence, apoptosis, autophagy and differentiation (60–63).

**FIGURE 1:**
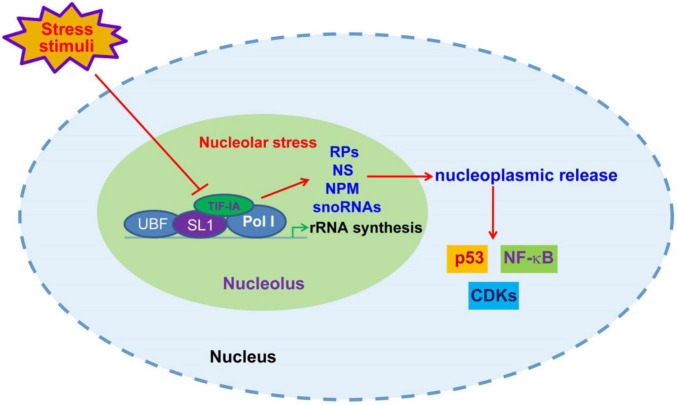
Nucleolar stress-initiated signaling transduction. Upon unfavorable stress stimuli, including DNA damages, nutrient deprivation, hypoxia and oxidative stress, the disruption of rRNA synthesis through posttranscriptional modification of Pol I transcriptional machinery components causes nucleolar malfunction and stress, leading to nucleoplasmic release of ribosomal proteins (RPs), NS, NPM and snoRNAs, and consequent modulation of p53, NF-κB and CDKs.

In addition to mdm2-p53 pathway, nucleolar stress also triggers stress events through p53-independent mechanisms (57, 64). Nucleolus and nucleolar stress have been reportedly involved in the regulation of a growing number of signaling pathways, such as NF-κB, HIF-1α and CDK4/6 (Figure 1) (20, 65, 66). It is speculated that nucleolar stress may participate in the regulation of a majority of non-ribosomal nucleolar functions discussed in section 2. A variety of nucleolar stress-inducing stimuli, such as hypoxia, heat shock and oxidative stress, cause altered distribution and function of nucleolar proteins and snoRNAs (55, 67). Accordingly, the pivotal role of the nucleolus in the development of various human diseases has increasingly attracted research and therapeutic attention in recent years. While the role of nucleolar stress in some chronic diseases, such as cancer and neurodegenerative diseases, has been intensively investigated, the connection between nucleolar stress and cardiovascular disease remains largely obscure (68). Moreover, previous studies suggest that nucleolar stress appears to elicit both protective and detrimental roles in cardiovascular disorders. These facts indicate a complicated role of the nucleolus in the development of cardiovascular diseases.

## The protective role of nucleolus in stress-induced cardiac malfunction

**FIGURE 2:**
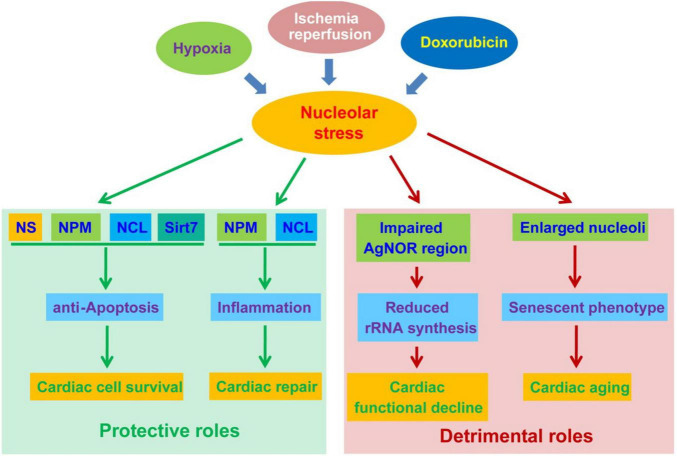
The protective and detrimental roles of nucleolar stress in cardiac disease progression. Nucleolar stress, as a result of hypoxia, ischemia-reperfusion (IR) and doxorubicin exposure, function as a double-edged sword in the development of cardiac diseases. On the one hand, nucleolar stress triggers the upregulation and altered distribution of cardioprotective nucleolar proteins. These proteins exert pro-survival functions in cardiomyocytes, and meanwhile activate inflammatory responses to promote cardiac repair. On the other hand, nucleolar stress may be associated with impaired rRNA transcription and enlarged nucleoli during the progression of cardiac diseases, leading to cardiac functional decline and aging.

Multiple nucleolar proteins, such as nucleostemin (NS), nucleophosmin (NPM) and nucleolin (NCL), possess pro-survival and pro-renewal properties (69, 70). Their expression and activity are of great importance to the fate decision of cardiomyocytes. Pioneer works by Mark Sussman’s lab revealed that the induction of nucleolar proteins, including NS and NPM, is an early event following cardiomyopathic injury (71, 72). The upregulation of NS and NPM not only occurred following exposure to nucleolar stress inducer doxorubicin and actinomycin D (ActD), but also was observed in hypoxia/hypoxia-reperfusion conditions. Given the fact that these proteins are highly expressed in cancer and stem cells and elicit pro-survival and pro-repair roles, their upregulation are supposed to prevent the injury and death of cardiac cells. As predicted, forced expression of either NS or NPM inhibit caspase activation and cardiac cell death. The opposite is also true, as depletion of NS or NPM significantly enhance damage-induced cell death (72). Notably, NS and NPM also displayed apparent nucleoplasmic distribution following stress stimuli, suggesting an initiation of nucleolar stress in cardiac cells. In line with these findings, our recent RNA-seq data confirmed that NS is significantly upregulated in hypoxia-conditioned cardiac progenitors (Unpublished data). It is worth of notice that p53 activation was only mildly observed in these conditions, potentially as a result of NS-mediated stabilization of mdm2 in the nucleoplasm (73). More recent data uncovered that NPM is also secreted into the extracellular space via an autophagy-dependent mechanism in human cardiac mesenchymal progenitors (74). Extracellular NPM functions as a ligand of TLR4 to initiate TLR4/NF-κB inflammatory response, potentially facilitating cardiac repair (75). These studies implicated protective roles of NS and NPM in the regulation of the survival and repair of cardiomyocytes (Figure 2).

In addition to NS and NPM, NCL also exhibits cardioprotective function during the progression of cardiomyopathy. Proteomic data revealed that NCL has higher chromatin-binding in the mouse heart during hypertrophy and failure (76). NCL was subjected to protein cleavage in the very early phases of cardiac ischaemia-reperfusion injury (77). Further investigations indicated that NCL expression was dramatically reduced in the early stage of experimental myocardial infarction, and then recovered at late stages (78). Overexpression of NCL protects cultured cardiomyocytes from hypoxia- and H_2_O_2_-induced death (77). These studies demonstrated that NCL may facilitate the recovery of cardiomyocytes following exposure to stress stimuli. Moreover, NCL has been reportedly implicated in inflammatory induction in experimental myocardial infarction, as well as in cultured cardiomyocytes following hypoxia-reoxygenation injury (78, 79). NCL plays a key role in the induction of a variety of cytokines important for inflammatory responses and cardiac repair, such as IL-6 and IL-1β (79). *In vivo* data revealed that NCL expression is critical for alternative (M2) polarization of macrophages, and depletion of NCL reduced M2 polarization but had no effect on general macrophage infiltration (78). Given the fact that macrophage M2 polarization is of vital importance to cardiac tissue repair following injury, it is anticipated that NCL expression may critically contribute to cardiac recovery via the initiation of alternative inflammatory responses (80).

Sirtuin 7 (Sirt7) is mainly localized in the nucleus, where it regulates rRNA transcription and nucleolar homeostasis via its deacetylase activity (81). Up on nucleolar stress, Sirt7 is released from the nucleolus to modulate stress-related signaling (82, 83). Being a member of sirtuin family proteins, Sirt7 has been implicated in the regulation of tissue repair, aging and metabolism. Similarly with NS and NPM, Sirt7 is upregulated in the early stages of acute cardiovascular injury (84). Sirt7 knockout increased the risk of cardiac rupture following myocardial infarction as a result of impaired activation of transforming growth factor-β (TGF-β) pathway. Another study demonstrated that Sirt7 ablation led to the development of heart hypertrophy and inflammatory cardiomyopathy, likely through the deacetylation and inactivation of p53 (85). These studies suggested that Sirt7 has emerged as a cardioprotective nucleolar protein.

Overall, these findings provide conceivable evidence indicating that nucleolar stress is initiated in cardiac cells following acute cardiovascular injury, which leads to the release of cardioprotective nucleolar proteins into nucleoplasm, where they may elicit cardioprotective function via diverse mechanisms, such as counteracting p53-mediated apoptosis. In addition to nucleoplasmic release, nucleolar proteins are also upregulated following cardiovascular injury, and participate in the repair and survival of cardiac cells. These data provide convincing demonstration of a cardioprotective role of nucleolus and nucleolar proteins in acute cardiomyopathy.

## The potential role of nucleolar stress in cardiac aging and functional decline

The initiation of nucleolar stress triggers various types of stress responses via stress-responsive pathways, especially mdm2-p53 pathway (86, 87). Depending on cell lineages, nucleolar stress has been associated with the induction of cell apoptosis, senescence, autophagy and differentiation. In cardiac cells, the cellular responses following nucleolar stress remain largely elusive. While nucleolar stress has been shown to inhibit apoptotic death of cardiomyocytes following myocardial damage, it is unclear how nucleolar stress influences cardiac cell fate decision under stress conditions (72). Particularly, nucleolar stress-inducing stimuli, such as hypoxia, ischemia/reperfusion and doxorubicin treatment, have been shown to facilitate the senescence of cardiomyocytes (88–90). It raises the question whether there is a link between nucleolar stress and senescent phenotype under these stress conditions.

Accumulating data have indicated a critical involvement of nucleolus and nucleolar stress in cell senescence and tissue aging. Genetic or pharmacological induction of nucleolar stress has been reported to trigger cell senescence and tissue degeneration (91, 92). Aberrant nucleolar morphology has been well-recognized as a hallmark of cell senescence, suggesting a mechanistic link between nucleolar function and senescence induction (93). In this regard, it is speculated that nucleolar stress may facilitate myocardial aging, in spite of a protective role during the early stages of acute myocardial damage (72). Notably, multiple nucleolar stress-inducing stimuli, including doxorubicin exposure, hypoxia-reoxygenation and ischemia-reperfusion, have been shown to facilitate the senescence of cardiomyocytes (89, 90, 94). Interestingly, cardiomyocyte senescence is partially attributed to the activation of the p53 pathway, implicating a link between nucleolar stress induction and p53-driven senescence in cardiomyocytes (89, 95). Currently, the evidence linking nucleolar stress and cardiac cell senescence is limiting. Heterozygous deletion of NS has been shown to induce the senescence of cardiac progenitors, suggesting that disturbing nucleolar proteins induces cardiac senescence (96). Disruption of rRNA transcription through TIF-IA ablation causes p53 accumulation and senescent phenotypes in smooth muscle cells (91). Whether similar phenotypes can be observed in cardiac cells is worthy of further investigations.

Studies also suggested that nucleolar disorder is correlated with cardiac functional decline. An early clinical study revealed that cardiomyocytes from patients with severe ischaemic heart disease complicated with heart failure displayed significantly reduced silver-stained nucleolar organiser regions (AgNORs) (97). AgNORs are mainly confined to the fibrillar center, where some nucleolar proteins can be specifically stained by silver nitrate (98, 99). Given the fact that the fibrillar center is responsible for rRNA transcription, the reduction of AgNOR suggests a negative correlation between nucleolar rRNA transcription and the severity of ischaemic heart disease (100). Because aberrant nucleolar function has been associated with genomic instability, the decline of nucleolar staining may potentially be indicative of increased genotoxic stress in cardiomyocytes (101, 102). In contrast to reduced nucleolar staining, NCL expression and overall size of nucleolus were increased both in ischemic and dilated cardiomyopathy (103). The mechanism underlying this difference remains unclear. Of note, enlarged nucleoli are widely regarded as a hallmark of senescence and aging (93). On the other hand, studies suggested that tissue aging is associated with hypermethylation and reduced transcriptional activity of rDNA (53, 104). Thus, the morphological changes observed in these studies may be associated with aging-like functional decline of cardiomyocytes (Figure 2). The causal relationship between nucleolar change and cardiac aging requires further mechanistic studies.

## Conclusion

While nucleolar stress has been implicated in assorted human diseases, its role in cardiovascular diseases received insufficient attention. Recent data obtained from pathological, animal model and cell culture studies all point to a critical involvement of nucleolar stress in the development of cardiovascular diseases. Moreover, different from its role in most other diseases, nucleolar stress is supposed to play both beneficial and detrimental roles in the progression of cardiovascular disease. In early stages of cardiac injury, nucleolar stress leads to the upregulation of various cardioprotective nucleolar proteins, such as NS, NPM, and NCL. The expression of these proteins plays a key role in facilitating the survival of cardiomyocytes. On the other side of the coin, cardiac functional decline and failure are correlated with apparent nucleolar abnormalities, including reduced AgNORs and enlarged nucleoli. It remains unclear whether these nucleolar alterations are merely a consequence of cardiomyopathy, or may contribute actively to the injury and death of cardiac cells. A deeper understanding of nucleolar alterations will undoubtedly provide novel insights into the pathogenesis of cardiac diseases.

Because of the complicated roles of the nucleolus in cardiac diseases, nucleolar intervention may be a promising strategy to protect against acute and chronic cardiac injury. Of importance, current data suggest that acute cardiac injury mostly initiate rapid upregulation of cardioprotective nucleolar proteins to facilitate the survival and repair of cardiomyocytes. However, chronic damage may profoundly impair rRNA transcription and nucleolar function, probably through impacting the epigenetic landscape of rDNA. These alterations within the nucleolus are tightly associated with cardiac aging and functional decline. We speculate that there are connections between of protein and epigenetic alterations in the nucleolus, given the fact that the nucleolus and nucleolar stress play a crucial role in epigenetic modulation (101). These discoveries suggest that both proteomic and epigenetic changes are involved in cardiac disease progression. In other words, intervening strategies both at protein and epigenetic levels may be beneficial to the amelioration of cardiac diseases. While upregulating the expression of nucleolar proteins has been proven to be feasible approaches, other therapeutic strategies, such as epigenetic approaches, remain to be developed. Moreover, the activities of nucleolar proteins, including NS, NPM and NCL, are also regulated at posttranscriptional levels, providing an additional option of therapeutic intervention. Taken together, we speculate that nucleolar intervention is a potential therapeutic strategy against the progression of cardiac diseases.

## Author contributions

DY: conducting the original draft preparation. LH: reviewing and editing the manuscript. Both authors contributed to the article and approved the submitted version.
